# Two-photon microscopy of Paneth cells in the small intestine of live mice

**DOI:** 10.1038/s41598-018-32640-7

**Published:** 2018-09-21

**Authors:** Won Hyuk Jang, Areum Park, Taejun Wang, Chan Johng Kim, Hoonchul Chang, Bo-Gie Yang, Myoung Joon Kim, Seung-Jae Myung, Sin-Hyeog Im, Myoung Ho Jang, You-Me Kim, Ki Hean Kim

**Affiliations:** 1 Division of Integrative Biosciences and Biotechnology, Pohang University of Science and Technology (POSTECH), 77 Cheongam-ro, Nam-gu, Pohang, Gyeongbuk 37673 Republic of Korea; 2 507 Avison Biomedical Research Center, Severance Biomedical Research Institute, Yonsei University College of Medicine, 50-1 Yonsei-ro, Seodaemun-gu, Seoul, 03722 Republic of Korea; 3 Department of Ophthalmology, University of Ulsan College of Medicine, Asan Medical Center, 88 Olympic-ro, 43-gil, Songpa-gu, Seoul, 05505 Republic of Korea; 4 Department of Gastroenterology, University of Ulsan College of Medicine, Asan Medical Center, 88 Olympic-ro, 43-gil, Songpa-gu, Seoul, 05505 Republic of Korea; 5 Academy of Immunology and Microbiology, Institute for Basic Science (IBS), 77 Cheongam-ro, Nam-gu, Pohang, Gyeongbuk 37673 Republic of Korea; 6 Graduate School of Medical Science and Engineering, Korea Advanced Institute of Science and Technology, 291 Daehak-ro, Yuseong-gu, Daejeon, 34141 Republic of Korea; 7 Department of Mechanical Engineering, Pohang University of Science and Technology (POSTECH), 77 Cheongam-ro, Nam-gu, Pohang, Gyeongbuk 37673 Republic of Korea

## Abstract

Paneth cells are one of the principal epithelial cell types in the small intestine, located at the base of intestinal crypts. Paneth cells play key roles in intestinal host-microbe homeostasis via granule secretion, and their dysfunction is implicated in pathogenesis of several diseases including Crohn’s disease. Despite their physiological importance, study of Paneth cells has been hampered by the limited accessibility and lack of labeling methods. In this study, we developed a simple *in vivo* imaging method of Paneth cells in the intact mouse small intestine by using moxifloxacin and two-photon microscopy (TPM). Moxifloxacin, an FDA-approved antibiotic, was used for labeling cells and its fluorescence was strongly observed in Paneth cell granules by TPM. Moxifloxacin labeling of Paneth cell granules was confirmed by molecular counterstaining. Comparison of Paneth cells in wild type, genetically obese (*ob*/*ob*), and germ-free (GF) mice showed different granule distribution. Furthermore, Paneth cell degranulation was observed *in vivo*. Our study suggests that TPM with moxifloxacin labeling can serve as a useful tool for studying Paneth cell biology and related diseases.

## Introduction

The small intestine is where most of the digestion and absorption of food takes place in the body. The intestinal epithelium also serves as a protective barrier against numerous microorganisms in the intestinal lumen and is composed of several cell types such as absorptive enterocytes, goblet cells, enteroendocrine cells, intestinal stem cells, and Paneth cells^1,2^. Paneth cells are terminally differentiated cells residing at the base of intestinal crypts, interspersed with intestinal stem cells. Paneth cells are found only in the small intestine, but not in the colon, of healthy individuals. Paneth cells have densely packed secretory granules containing antimicrobial factors such as lysozyme (Lyz) and antimicrobial peptides (AMPs) for mucosal defense, and play key roles in maintaining intestinal homeostasis against commensal and pathogenic microorganisms via degranulation^2–4^. Paneth cells also help to sustain and modulate intestinal stem cells by secreting essential growth factors and regulatory molecules^5^. Deficiency or dysfunction of Paneth cells, therefore, is associated with microbial dysbiosis, tissue regeneration failure, and various inflammatory diseases in the gut^6–9^. On the other hand, severe inflammation and microbial imbalance caused by environmental stressors or vulnerable genetic predisposition can lead to functional alteration or apoptosis of Paneth cells. Moreover, presence of Paneth cells outside of the small intestine, called Paneth cell metaplasia, is often observed in patients with colitis and other gastrointestinal inflammatory diseases^10,11^.

Historically, studies of Paneth cells mainly focused on their antimicrobial functions. Their studies had to rely on a short-term intestinal crypt culture or the immunostaining of *ex vivo* tissues, because isolated Paneth cells did not survive in culture conditions. With recent advances in the intestinal organoid culture, long-term studies of Paneth cells are now possible, rendering molecular and cell biological dissection of Paneth cell functions much more feasible^5^. Despite the numerous advantages, however, the intestinal organoid culture system comprised only of epithelial cells is short of recapitulating the intricate cross-talks among epithelial cells, immune cells, stromal cells, and nerve cells that are present in the intact small intestine. Thus, it is highly desirable to develop a reliable method to study Paneth cells *in situ* in live animals.

With the advance of microscopic techniques such as two-photon microscopy (TPM), intravital imaging has been used to study various animal organs including the mouse small intestine^12–15^. TPM is a nonlinear fluorescence microscopic technique, capable of three-dimensional (3D) cellular imaging of live organs with its relatively high-imaging depths and reduced photodamage^16,17^. Distribution and behavior of immune cells in the small intestine were studied by TPM with either immunofluorescent staining or transgenic (Tg) mice expressing fluorescent proteins^12,13^. Label-free TPM based on the intrinsic contrasts such as autofluorescence (AF) and second harmonic generation (SHG) was also used to image the intestine^14,15^. Recently, we introduced moxifloxacin as a non-specific cell-labeling agent for TPM^18,19^. Moxifloxacin is an FDA-approved antibiotic for the treatment or prevention of ocular and pulmonary infections and has excellent tissue penetration characteristics^20,21^. Moxifloxacin has an intrinsic fluorescence property and its two-photon (TP) fluorescence was characterized^18,22^. TPM of biological tissues with topical application of moxifloxacin ophthalmic solution showed approximately 10-fold fluorescence enhancement of cells compared to AF^19^.

Herein, we demonstrate a new *in vivo* imaging method of using moxifloxacin and TPM for observing Paneth cells and their granules in the intact mouse small intestine. Distinctive granular structures of Paneth cells were clearly visible at the base of intestinal crypts when moxifloxacin-based TPM was used to image the small intestine from the serosa. Moxifloxacin labeling of Paneth cell granules was verified by counterstaining with specific fluorescent markers. Paneth cells of various mouse types such as wild type mice and genetically obese (*ob/ob*) mice raised in conventional specific pathogen-free (SPF) condition, and wild type mice raised in germ-free (GF) condition were imaged and compared. Furthermore, Paneth cell degranulation was monitored by time-lapse TPM imaging *in vivo*.

## Results

### Moxifloxacin-based TPM of Paneth cells and their granules in the small intestine of live mice

**Figure 1:**
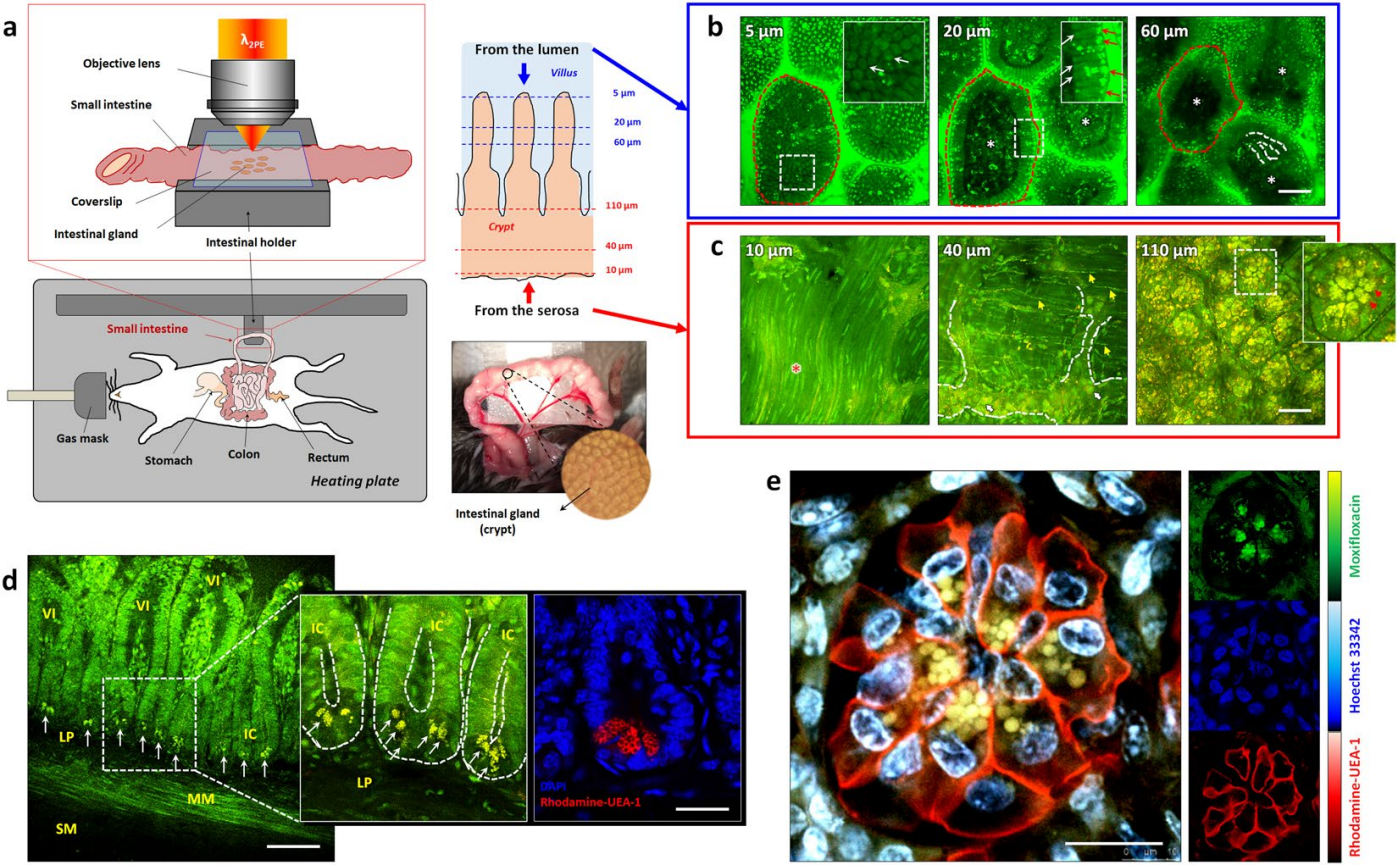
*In vivo* TP images of the mouse small intestine with moxifloxacin labeling. (**a**) TPM setup for *in vivo* imaging of the anesthetized mouse small intestine. A portion of the small intestine was gently pulled out from the abdominal cavity, held on the temperature-controlled moisture-maintained intestinal holder, and sealed with a coverslip. (**b**) Moxifloxacin-based 3D TPM images taken from the luminal side of the small intestine in a wild type mouse (Supplementary Video 1). 3D TPM images revealed well-organized enterocytes (white-arrows) on the surface of the villi (red-dashed-circles), some cells inside the villi (white-asterisks), and vascular structures inside the villi (white-dashed-line), and strong moxifloxacin fluorescence expressing microstructures (red arrows in inset), which are likely lysosomes in enterocytes. (**c**) Moxifloxacin-based 3D TPM images taken from the serosal side of the small intestine in a wild type mouse (Supplementary Video 2). 3D TPM images showed the outer muscle (red-asterisks), myenteric plexus (white-dashed-lines), fibrous structures, (yellow-arrow), and glial cells (white-arrows) at the bottom of intestinal crypts. In the relatively deep region from the serosal surface, Paneth cells with multiple, clustered granules (red arrowheads in inset) were detected at the base of intestinal crypts. (**d**) Moxifloxacin-based cross-sectional TP images of the *in vivo* small intestine and *ex vivo* cross-sectional fluorescence image of the sectioned small intestine tissue of wild type mice (Supplementary Video 3). TP images showed cross-sectional structures of the small intestine including intestinal villi (VI), intestinal crypts (IC), lamina propria (LP), muscularis mucosa (MM), and submucosa (SM). Bright granular structures (white-arrows) were visible at the base of intestinal crypts owing to the stronger expression of moxifloxacin fluorescence compared with other subcellular structures. Corresponding fluorescence image of the sectioned small intestinal tissue showed the location and the morphologies of the Paneth cell granules at the epithelial lining of the small intestine. DAPI (*blue*) and rohdamine-UEA-1 (*red*) stained nuclei and Paneth cell granules, respectively, in the fixed and permeabilized small intestinal section. (**e**) Counterstained TPM images of the small intestinal crypt base of a live mouse (Supplementary Video 4). Cells containing spherical-shaped granules, highly labeled with moxifloxacin fluorescence (*green*), were confirmed to be Paneth cells by counterstaining the cell membrane with rhodamine-UEA-1 (*red*). Cell nuclei were labeled with Hoechst 33342 (*blue*). Individual fluorescence signal from each fluorescence contrast agents are displayed in small subsets (right panel, moxifloxacin: *green*, Hoechst 33342: *blue*, rhodamine-UEA-1: *red*). In this experiment, the number of mice used are as follows; wild type C57BL/6 mice bred in a SPF condition (n = 5). Scale bars, (**b**,**c**): 50 µm; (**d**), 100 µm and 15 µm; (**e**), 15 µm.

*In vivo* moxifloxacin-based TPM of the small intestine in wild type C57BL/6 specific-pathogen-free (SPF) mice was conducted by using an intestinal holder (Fig. 1a). The mouse was anesthetized using respiratory anesthesia and an incision was made on the abdomen to access the small intestine. The small intestine was gently pulled out from the abdominal cavity and held on the temperature-controlled intestinal holder (Supplementary Fig. 1). Moxifloxacin ophthalmic solution was topically administered on either luminal or serosal side the small intestine several minutes before TPM and it quickly penetrated tissues owing to its high aqueous solubility and lipophilicity^20^. For TPM imaging from the luminal side, a 5 mm longitudinal incision was made on the small intestine to expose the lumen. 3D TPM images of the small intestine from the lumen showed epithelial cells on the surface of the villi, while vasculatures and other cells were detected inside the villi (Fig. 1b and Supplementary Video 1). Since relatively small excitation power was used for moxifloxacin-based TPM, the AF signal could be negligible. Moxifloxacin seemed to label most of cells with varying degrees, but with no clear specificity. Certain cell types such as absorptive enterocytes on the top of villi, and immune cells inside the villi could be identified based on their spatial locations and morphologies. 3D TPM images of the small intestine from the serosa showed various structures including the muscle, myenteric plexus, fibrous structures and intestinal crypts (Fig. 1c and Supplementary Video 2). Especially, spherical granules densely distributed at the base of intestinal crypts were clearly visible owing to the strong moxifloxacin fluorescence. Cross-sectional TPM images from the incision surface showed that these granules were apically located at the base of epithelial linings (Fig. 1d and Supplementary Video 3). They were considered to be Paneth cell granules, because Paneth cells are the only granule-containing cells located at the base of intestinal crypts. The apical localization of Paneth cell granules were confirmed by staining the *ex vivo* small intestinal tissue section with rhodamine-conjugated *Ulex Europaeus agglutinin 1* (UEA-1). UEA-1 specifically binds to glycoproteins containing α-linked fucose that are highly enriched in Paneth cell granules and also present in Paneth cell membrane. To ascertain that the moxifloxacin-labeled granular structures indeed reside inside Paneth cells, the intact small intestine was labeled with moxifloxacin, rhodamine-UAE-1, and Hoechst 33342 (Fig. 1e and Supplementary Video 4). *In vivo* TPM revealed that the moxifloxacin-labeled granules were enclosed within UEA-1-positive Paneth cell membrane. Spectral unmixing was applied to separate individual fluorescent contrast agents, and these were displayed in pseudo-colors (*blue*: Hoechst 33342, *green*: moxifloxacin, *red*: rhodamine-UAE-1) in TPM images. The spectrally unmixed blue, green, and red channel images showed cell nuclei labeled with Hoechst 33342, granular structures labeled with moxifloxacin, and Paneth cell membranes labeled with rhodamine-UAE-1, respectively. The cells intercalated in between Paneth cells containing moxifloxacin-labeled granules were considered to be intestinal stem cells, based on their well-understood spatial distribution. Further investigation with Tg mice having a fluorescent marker for *Lgr*5 would conclusively identify intestinal stem cells^5,23^.

### Comparison of Paneth cell granules in mice under different environmental and metabolic conditions

*In vivo* moxifloxacin-based TPM could visualize Paneth cell granules in the small intestine of wild type SPF mice. Since Paneth cells play central roles in maintaining the intestinal host-microbial homeostasis, different intestinal environments might be associated with alterations of Paneth cell granules^24^. Previous immunohistochemical analysis of the small intestinal sections showed reduced lysozyme contents of Paneth cell granules in obese humans and animals compared to lean controls^25,26^. Therefore, we decided to test whether moxifloxacin-based TPM could be used for detecting the alterations of Paneth cell granules in live mice under different environmental and metabolic conditions.

**Figure 2:**
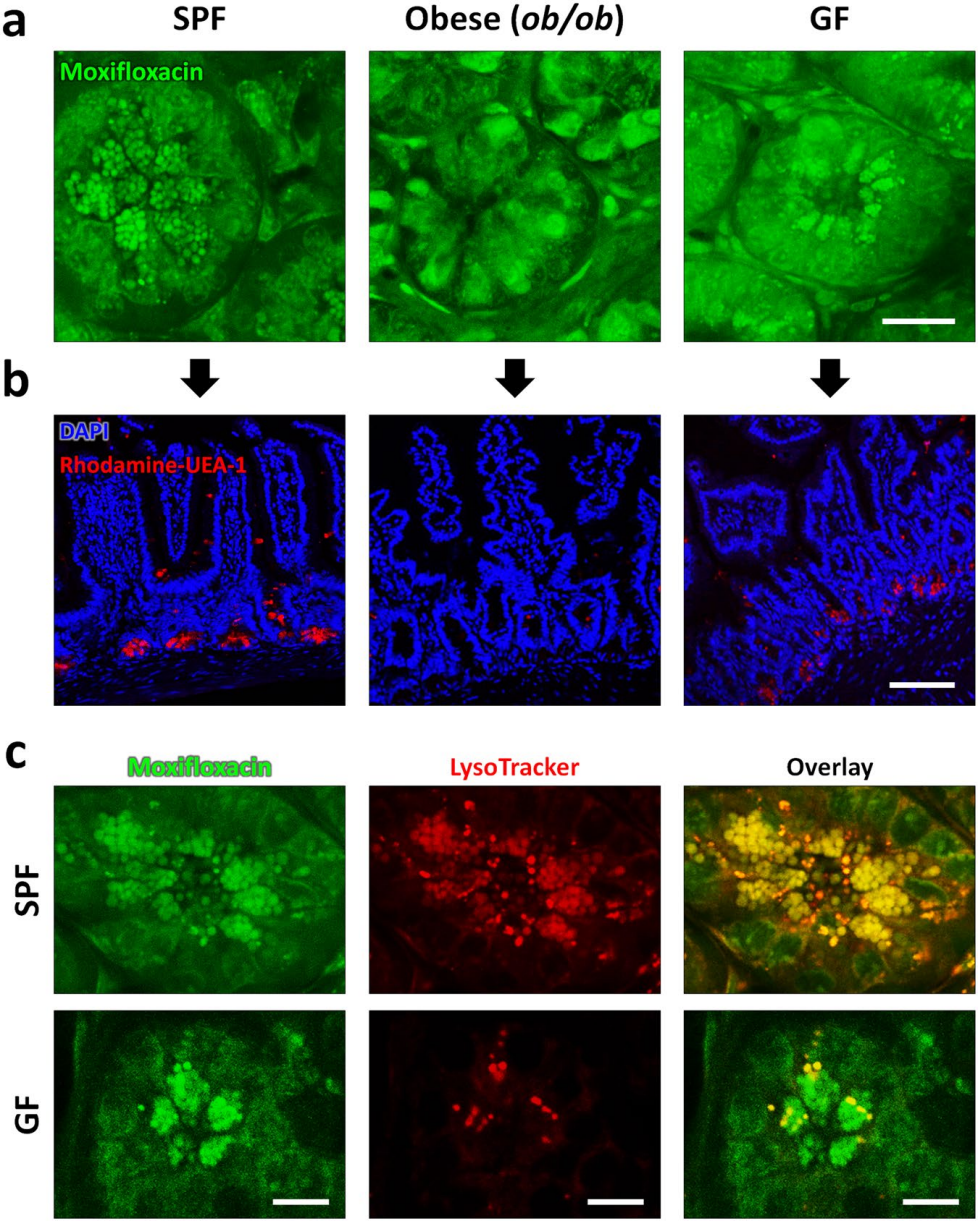
*In vivo* Moxifloxacin-based TPM images and *ex-vivo* fluorescently labeled images of small intestinal crypts in mice under different environmental and metabolic conditions. (**a**) *In vivo* moxifloxacin-based TPM images at the base of intestinal crypts in SPF, obese (*ob/ob*), and GF mice. In wild type SPF mice, Paneth cell granules are clearly visible via moxifloxacin labeling. In obese (*ob/ob*) mice, Paneth cell granules did not appear at the base of intestinal crypts, but there were some cells expressing moxifloxacin fluorescence in the cytoplasm. In wild type GF mice, Paneth cell granules appeared similarly to those of SPF mice in moxifloxacin-based TPM. (**b**) *Ex vivo* fluorescence images of the small intestine sections from SPF, obese (*ob/ob*), and GF mice. Cell nuclei and Paneth cell granules in the fixed and permeablilized small intestinal sections were stained with DAPI (*blue*) and rhodamine-UEA-1 (*red*), respectively. Fluorescence images of the sectioned small intestine tissues showed epithelial linings and Paneth cell granules at the bottom. Paneth cell granules were visible in SPF and GF mice only, and not detected in obese (*ob/ob*) mice. These fluorescence image results of the sectioned intestinal crypts were consistent with *in vivo* moxifloxacin-based TPM results. (**c**) *In vivo* TPM images of Paneth cell granules in SPF and GF mice with the counterstaining of both moxifloxacin and LytoTracker. TPM images of SPF mice showed that both moxifloxacin and LysoTracker similarly labeled Paneth cell granules. On the other hand, TPM images of GF mice showed that LysoTracker labeled only a portion of moxifloxacin-labeled Paneth cell granules. In this experiment, the number of mice used are as follows; wild type C57BL/6 mice bred in a SPF condition (n = 5), germfree (GF) condition (n = 9), obese (ob/ob) mice (n = 8). Scale bars in (**a**,**b**), and (**c**) are 20 μm, 100 μm, and 10 μm, respectively.

Paneth cells in wild type C57BL/6 mice raised in a GF condition (GF) and genetically obese (*ob*/*ob*) mice raised in a SPF condition were compared to Paneth cells in lean, wild type C57BL/6 mice raised in a SPF condition (SPF). TPM with and without moxifloxacin labeling was conducted in these mice. TPM without moxifloxacin labeling was based on the intrinsic AF of cells and the higher excitation power was used than the one for TPM with moxifloxacin labeling (Supplementary Fig. 2, Supplementary Videos 6,8,10). Moxifloxacin-based TPM images taken *in vivo* (Fig. 2a) were compared with fluorescence images of the sectioned small intestinal tissues stained with rhodamine-UAE-1 (*red*) and DAPI (*blue*) for labeling Paneth cell granules and the cell nucleus, respectively (Fig. 2b). In SPF mice, moxifloxacin-based TPM image visualized densely distributed Paneth cell granules at the base of intestinal crypts (Supplementary Video 5), and fluorescence images of the small intestinal sections confirmed the distribution of Paneth cell granules. Compared to lean SPF mice, obese (*ob*/*ob*) mice showed almost no granular structures. Instead, relatively high moxifloxacin fluorescence was detected in some cells (Supplementary Video 7). Fluorescence images of the small intestine sections from obese (*ob*/*ob*) mice also revealed comparable results with the moxifloxacin-based TPM images, showing few or no granules at the base of intestinal crypts. Our *in vivo* moxifloxacin-based TPM imaging results correlated well with the previous findings that lysozyme-containing Paneth cell granules are significantly reduced in obese individuals^25^. Collectively, *in vivo* moxifloxacin-based TPM of obese (*ob*/*ob*) mice showed quite different states of Paneth cells from those in lean SPF mice. In GF mice, moxifloxacin-based TPM images clearly showed apically clustered Paneth cell granules at the base of intestinal crypts (Supplementary Video 9). The distribution pattern of Paneth cell granules in GF mice was not much different from that in SPF mice. Fluorescence images of the sectioned small intestinal tissues confirmed the distribution of Paneth cell granules in GF mice. Although moxifloxacin-based TPM showed similar distribution of Paneth cell granules in both SPF and GF mice, a previous report showed a different composition of Paneth cell granules in GF and SPF mice^24^. Because the composition of granule contents likely affects the pH of Paneth cell granules, we used LysoTracker to counterstain Paneth cell granules together with moxifloxacin in both SPF and GF mice (Fig. 2c). LysoTracker is a fluorescent dye that preferentially accumulates in intracellular organelles having low pH, including late endosomes, lysosomes, and some secretory granules. Fluorescence from moxifloxacin and LysoTracker was spectrally resolved and color-coded in *green* and *red*, respectively. In SPF mice, TPM images in both moxifloxacin and LysoTracker channels showed almost same distribution of Paneth cell granules. An overlaid TPM image of the two channels showed good co-registration in most of granules, although there were slight intensity variations in between *green* and *red* channels. However, in GF mice, TPM images showed much fewer LysoTracker-labeled granules than moxifloxacin-labeled granules. Partial LysoTracker labeling of Paneth cell granules in GF mice might be associated with heterogeneous Lyz contents in Paneth cell granules, as previously suggested^24^. More granule labeling by moxifloxacin than LysoTracker in GF mice might indicate that moxifloxacin labeling is less affected by the granule composition or the pH condition than LysoTracker labeling. The exact mechanism of moxifloxacin labeling of Paneth cell granules is yet unknown, however a hypothesis is presented in Discussion section. In addition to moxifloxacin-based TPM imaging, TPM imaging without moxifloxacin was conducted to visualize intestinal crypts based on intrinsic AF and SHG (Supplementary Fig. 2). TPM without moxifloxacin could not visualize Paneth cell granules in all the SPF, obese (*ob*/*ob)*, and GF mice, probably because they do not express AF. However, TPM without moxifloxacin showed different AF expression of cells at the base of intestinal crypts in these mouse conditions. In wild type SPF mice, some cells at the base of intestinal crypts were visible by expressing relatively strong AF. However, in both obese (*ob*/*ob)* and wild type GF mice, cells were difficult to visualize based on AF. Different AF expression in cells might be associated with either different environmental and metabolic conditions. Although moxifloxacin-based TPM visualized Paneth cell granules in both wild type SPF and GF mice, TPM without moxifloxacin showed different AF expression in the cells at the base of intestinal crypts. TPM both with and without moxifloxacin showed different states of Paneth cells and their granules in these mouse conditions.

### Observation of Paneth cell degranulation by using moxifloxacin-based TPM *in vivo*

**Figure 3:**
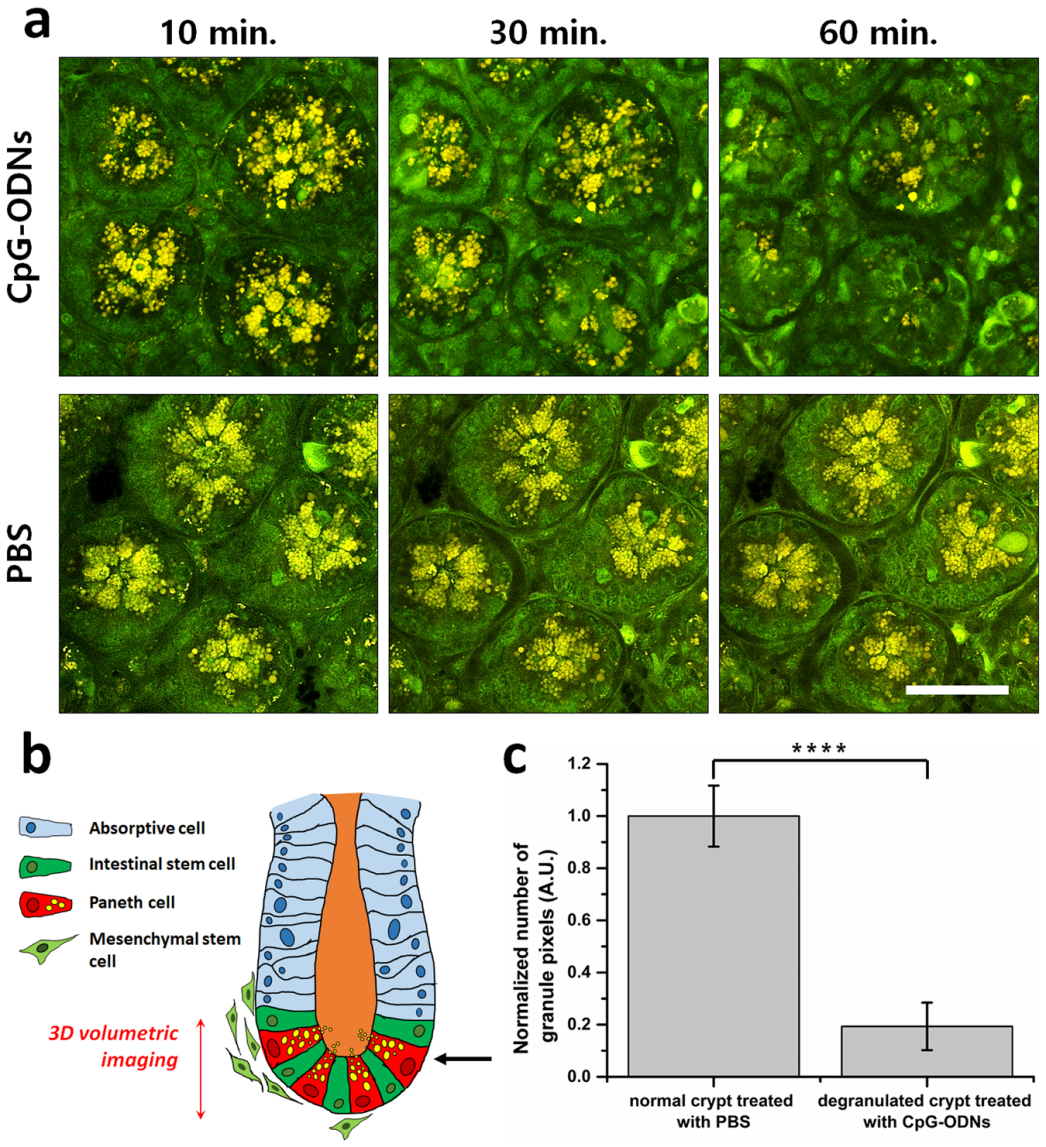
*In vivo* observation of Paneth cell degranulation in SPF mice using moxifloxacin-based TPM. (**a**) Longitudinal moxifloxacin-based TPM images of intestinal crypts at the base after intraluminal injection of CpG-ODNs (top panel, 10 µM), and PBS (lower panel) at different time points. Longitudinal TPM images are presented at single representative depth planes in 10 min, 30 min, and 60 min post-injection. (**b**) A schematic of the small intestine showing the depth range of 3D TPM (red-arrow), and single representative depth plane (black-arrow). (**c**) Quantitative analysis of total granule pixel counts in the moxifloxacin-based 3D TPM images of degranulated intestinal crypts in SPF mice in 5 hours after intraperitoneal injection of CpG-ODNs. Statistical analysis was performed by Welch’s t-test; ****p < 0.0001. In this experiment, the number of mice used are as follows; wild type C57BL/6 mice bred in a SPF condition (n = 5). Scale bars in (**a**) indicate 100 µm.

Moxifloxacin-based TPM was used to observe the degranulation of Paneth cells in wild type SPF mice. Paneth cell degranulation was induced by injecting CpG oligodeoxynucleotides 1826 (CpG-ODNs) into mice either intraluminally or intraperitoneally. CpG-ODNs mimic bacterial DNA and trigger exocytosis of large secretory granules in Paneth cells via TLR9 activation^2,27^. *In vivo* degranulation process of Paneth cells was monitored by longitudinal TPM after the intraluminal injection of CpG-ODNs. For the negative control, phosphate-buffered saline (PBS) solution in the equal amount was injected using the identical protocol. 3D TPM imaging was conducted longitudinally at the same sites of the mouse small intestine in 10 min, 30 min, and 60 min after the injection. The longitudinal representative single x-y plane TPM images of Paneth cells at the bottom of the intestinal crypts after the injection are shown (Fig. 3a). A schematic of the small intestine shows the depth range of 3D TPM and the depth location of the representative TPM images (Fig. 3b). In 10 minutes after the injection, Paneth cell granules in the CpG-ODN treated mouse were slightly spread out compared to those in the control mouse, but the total amount of granules in the CpG-ODN treated mouse was comparable to that of the control mouse. In 30 minutes after the injection, the reduction of granules was observed in moxifloxacin-based TPM image compared to the one in 10 minutes after the injection. In 60 minutes after the injection, there were almost no granules in moxifloxacin-based TPM image at the bottom of intestinal crypts. Degranulation appeared to be completed in 60 minutes after the injection at the observed site. Paneth cell degranulation was observed to occur only in approximately 10% of the intestinal crypts in the CpG-ODN treated mice. Therefore, the observation of degranulation process via longitudinal TPM of the same sites was quite difficult. For the quantitative and statistical analysis of Paneth cell degranulation by CpG-ODN treatment, Paneth cell degranulation was examined by single-time TPM imaging after the CpG-ODN treatment. Paneth cell degranulation was induced by intraperitoneal injection of CpG-ODNs into the wild type mice 5 hours prior to TPM. Then, intestinal crypts containing degranulated Paneth cells were searched and imaged in 3D by TPM after moxifloxacin labelling. Paneth cell degranulation was analyzed quantitatively by counting the total number of the granule pixels in 3D TP images of the intestinal crypts (Fig. 3c). The control crypts, which were treated with PBS, were analyzed the same way. The total granule pixel counts in the degranulated intestinal crypts of CpG-ODN treated mice were compared with those in the intestinal crypts of the control mice. The total granule pixel counts in the degranulated intestinal crypts of CpG-ODN treated mice were approximately 20% of those in the intestinal crypts of control mice, on average. Although the rate and extent of Paneth cell degranulation process might change depending on various factors such as the dose and administration route of CpG-ODNs, it was clear that Paneth cell degranulation could be monitored *in vivo* by using moxifloxacin-based TPM.

## Discussion

In this study, a new *in vivo* imaging method of Paneth cell granules in the intact mouse small intestine was developed and verified. TPM was used to assess Paneth cells in the small intestine of live mice. Moxifloxacin was used to label Paneth cell granules as well as other cells by topical administration. Although moxifloxacin labeled cells non-specifically, it labeled Paneth cell granules especially well with strong fluorescence in wild type SPF mice. Moxifloxacin labeling of Paneth cell granules was confirmed by counterstaining Paneth cells with an immunofluorescent marker, rhodamine-UEA-1 (Supplementary Fig. 3 and Supplementary Video 11). Moxifloxacin-based TPM was applied to mice in different environmental and metabolic conditions such as obese (*ob*/*ob*) and GF mice. In obese (*ob*/*ob*) mice, moxifloxacin-based TPM showed much less Paneth cell granules at the base of intestinal crypts. *In vivo* TPM results of obese (*ob*/*ob*) mice were confirmed by fluorescence images of the small intestine section stained with DAPI (*blue*) and rhodamine-UEA-1 (*red*), and was consistent with a previous study^25^. In GF mice, moxifloxacin-based TPM showed similar distribution of Paneth cell granules at the base of intestinal crypts to those of SPF mice. Moxifloxacin labeling of Paneth cell granules was examined further by counterstaining with LysoTracker. While LysoTracker labeled Paneth cell granules similarly to moxifloxacin in the case of SPF mice, it labeled only a small portion of the moxifloxacin-labeled Paneth cell granules in GF mice. LysoTracker labeling results of these two mouse conditions were consistent with the previous studies, which showed alteration of Paneth cell granule contents in GF mice compared with SPF mice^25,26^. Since moxifloxacin labeled more Paneth cell granules than LysoTracker in GF mice, moxifloxacin labeling might be less sensitive to granule compositions or conditions.

Although moxifloxacin seemed to label most cells in the mouse small intestine with varying degrees and no clear specificity in TPM, it labeled Paneth cell granules especially well compared to other cellular structures. Moxifloxacin labeling mechanism of Paneth cell granules might be explained at least partially by a decrease in moxifloxacin’s lipophilicity in acidic conditions^28,29^. Moxifloxacin can pass through the membrane of cells and granules owing to its relatively high lipophilicity. Once Moxifloxacin enters the granules, its lipophilicity could decrease in the acidic microenvironment, leading to its accumulation within the granules. However, moxifloxacin and LysoTracker counterstaining results showed that moxifloxacin labeling of Paneth cell granules could be less dependent on pH, suggesting that there might be environmental factors other than pH contributing to the retention of moxifloxacin inside Paneth cell granules.

Moxifloxacin-based TPM of Paneth cells showed slightly different fluorescence spectrum of moxifloxacin in granules compared to other parts such as the cell cytoplasm. Moxifloxacin fluorescence in Paneth cell granules was slightly red-shifted, and this might be related with the pH variation in various intracellular compartments. The single-photon fluorescence spectrum of moxifloxacin was measured in various pH conditions from 3.76 to 8.20 (Supplementary Fig. 4). Moxifloxacin fluorescence at relatively low pH such as 4 and 5 showed peak intensity at relatively longer wavelength compared to the one at relatively high pH such as 7.5 and 8.2 (Supplementary Fig. 4b). The shift of emission spectrum peak occurred between pH level 5 and 7. Below pH 5, the ratio of fluorescence intensity at 510 nm to the one at 460 nm was approximately 2.2. Above pH 7, the intensity ratio between the two wavelengths was approximately 0.5 (Supplementary Fig. 4c). Paneth cell granules are known to be acidic, and this could explain the red-shift of moxifloxacin emission spectrum in TPM images of Paneth cell granules (Fig. 3).

We induced Paneth cell degranulation in live mice either by intraluminal or intraperitoneal injection of CpG-ODNs. In the longitudinal imaging experiments, we observed Paneth cell degranulation in a relatively limited (10 ~ 20%) region of the small intestine. It is possible that CpG-ODN-induced Paneth cell degranulation might be a prolonged process and occur in a non-synchronized fashion among the cells in different regions of the small intestine. Interestingly, CpG-ODNs was unable to induce Paneth cell degranulation in an *in vitro* study using the intestinal organoids^30^. In the same study, only IFN-γ among various microbial components and cytokines was shown to induce Paneth cell degranulation *in vitro*. Because the intestinal organoids were made of only epithelial cells and devoid of immune cells and other cell types, those results suggest that CpG-ODN-induced Paneth cell degranulation is an indirect process and might require production of IFN-γ *in vivo*. Moreover, the IFN-γ-induced Paneth cell degranulation *in vitro* was not synchronous even among the cells within a single crypt of intestinal organoids, and the onset and completion of degranulation was variable between organoids and took up to 24 hours^30^. Therefore, it is plausible that the extent of Paneth cell degranulation by indirect stimulation with CpG-ODNs *in vivo* is intrinsically variable and depends on many factors such as the availability of IFN-γ-producing cells nearby. Comparison of kinetics and extents of Paneth cell degranulation in live mice after stimulation with various agents such as microbial components, cytokines and cholinergic agonists might help to better understand the mechanism of Paneth cell degranulation *in vivo*.

Moxifloxacin-based TPM could be applied to functional imaging of Paneth cells, aiding the study of intestinal homeostasis and various gut diseases such as Crohn’s disease and ulcerative colitis^1,9,11,31^. A recent study showed that Paneth cell dysfunction could be used as an early indicator of some diseases^11,32,33^. Notably, the potency, safety, and pharmacology of the moxifloxacin administration have been studied in various tissues^34,35^, and it was confirmed that moxifloxacin penetrates and accumulates in the gut^36^. Moxifloxacin-based TPM could assess Paneth cells and granules in the intact mouse small intestine by 3D imaging from the serosa. In mice, Paneth cells and granules were located at relatively shallow depth, approximately 80 µm deep from the serosa. However, Paneth cells in larger animal models might not be assessable due to increased tissue thickness. Multiphoton imaging methods using longer excitation wavelengths such as three-photon microscopy might be useful for assessing Paneth cells in large animal models with higher imaging depths than TPM^37,38^. Longitudinal TPM of the small intestine in the current setup was limited to approximately 8 hours, which may not be enough for some other *in vivo* applications. Longitudinal *in vivo* TPM for the longer time duration could be possible by using an abdominal window chamber^39,40^.

In this study, Paneth cells and granules in the intact mouse small intestine were visualized *in vivo* by TPM with moxifloxacin labeling. *In vivo* TPM with and without moxifloxacin labeling showed different states of Paneth cell granules at the base of intestinal crypts in SPF, obese (*ob*/*ob*), and GF mice. Moxifloxacin-based TPM would be useful for studying the interplay of small intestinal Paneth cells and mucosal immune defense system in health^3,41–43^, inflammatory bowel disease (IBD) pathogenesis^33,44–48^, and metabolic syndrome^49^. Perhaps, it could also be developed as a diagnostic or prognostic method including endoscopy to examine the abnormalities of Paneth cell granules in IBD animal models and human patients *in vivo*^33,45,46^. Therefore, TPM with moxifloxacin labeling might be useful to study Paneth cell functions and related diseases in preclinical animal models.

## Materials and Methods

### Moxifloxacin

Moxifloxacin ophthalmic solution (*Vigamox*, Alcon Laboratories, Fort Worth, US) was used during the experiment to label cells including Paneth cells in the small intestine of live mice. This solution contains 0.5% (5 mg/mL) moxifloxacin hydrochloride (12.4 mM, pH 6.8) as the active ingredient. Moxifloxacin ophthalmic solution was always kept enclosed to prevent possible photo-bleaching. Moxifloxacin solution was topically administered to label Paneth cells in the small intestine.

### Two-photon microscopy

Both a commercial TPM (TCS SP5 II MP, Leica) and a custom-built TPM were used in this study. The commercial TPM used a Ti-Sapphire laser (Chameleon Vision II, Coherent) with specifications of 140 fs pulse width and 80 MHz repetition rate and a 25× objective lens (HCX IRAPO L25×, NA 0.95 W, Leica). The custom-built TPM used another Ti-Sapphire laser (Chameleon Ultra II, Coherent) and a 20× objective lens (XLUMPLFLN-20XW, 1.0 NA, water immersion, Olympus, Japan). Laser power was measured at the back aperture of the objective lens using a power meter (S310C, Thorlabs Inc.), and the laser power at the sample was calculated by accounting the beam clipping at the back aperture of the objective lens and transmission efficiency of the objective lens. Excitation laser was tuned to 780 nm for both moxifloxacin and autofluorescence based TPM. Excitation laser power was approximately 5–15 mW and 50–120 mW depending on the imaging depth for moxifloxacin and autofluorescence based imaging, respectively. The commercial TPM had 4 non-descanned detection (NDD) PMT channels and was used for spectral imaging with either moxifloxacin only or additional fluorescence labeling. The custom TPM had 2 detection channels and was used for either autofluorescence or moxifloxacin-based imaging. In moxifloxacin-based TPM using the commercial TPM, emission light was spectrally resolved by 4 NDD channels consisting of a set of dichroic mirrors of 495 nm, 560 nm, 620 nm. The acquired images were displayed in 2 pseudo-colors of *green* (300–495 nm), and *red* (495–680 nm). In case of triple labeling with Hoechst 33342, moxifloxacin, and rhodamine-UEA-1, emission light was spectrally resolved by 4 NDD channels using the same filter sets as the above. The acquired images were displayed in 3 pseudo-colors of *blue* (300–495 nm), *green* (495–560 nm), and *red* (560–680 nm). In case of moxifloxacin and Lysotracker double labeling and TPM with the commercial system, the excitation wavelength was set at 800 nm, and emission fluorescence was spectrally resolved and displayed in 2 pseudo-colors (*green*: 300–560 nm for moxifloxacin, *red*: 565–680 nm for LysoTracker). Moxifloxacin-based imaging with the custom TPM was conducted with a single emission detection channel covering 300–680 nm and was displayed in *green* pseudo-color. Autofluorescence based imaging with the custom TPM used 2 detection channels and was displayed in different pseudo-colors (*blue*: 300–430 nm, *green*: 430–680 nm). 3D TPM was conducted by acquiring multiple x-y plane images with a stepwise increment of 2 μm in the z direction. TP images consisted of either 512 × 512 pixels or 1024 × 1024 pixels and had the field of view of either 300 μm × 300 μm, 150 μm × 150 μm, or 75 μm × 75 μm. Imaging speed was approximately 0.8 frames/s and 0.2 frames/s for the commercial and custom-built TPM systems, respectively.

### Mice

All mice were kept at the animal facility of POSTECH Biotech Center under a SPF or a GF condition. Wild type C57BL/6 mice (8–10 weeks-old) bred in a SPF condition (SPF mice, n = 15) or a GF condition (GF mice, n = 9) were used. Obese (*ob*/*ob)* mice (n = 8) in a C57BL/6 background were purchased (Japan SLC Inc., Japan), and kept in a SPF condition with a normal chow diet, and subjected to TPM experiments when the weight reached 40~50 g. All animal experimental procedures were conducted in accordance with institutional guidelines and regulations and approved by the Institutional Animal Care & Use Committee at Pohang University of Science and Technology (POSTECH-2017-0027). After each experiment, the mice were humanely euthanized.

### Preparation and labeling of the small intestine for *in vivo* TPM

Mice were anesthetized via a face mask administrating a gas mixture of 1.5%/vol isoflurane (Terrell^TM^, Primal) and medical grade oxygen. To perform *in vivo* TPM of the small intestine, a custom-built intestinal holder (Live Cell Instrument, Rep. of Korea) was used (Supplementary Fig. 1). The intestinal holder consisted of a holder with a groove to keep the pulled small intestine in position, a magnetically attached cover with an optical window to gently press the small intestine and to image though, a fluid channel to supply saline to the holder groove and to keep the small intestine moisturized. The pulled small intestine was kept in moist condition during *in vivo* TPM. The mouse body and extracted small intestine were kept at 37 °C by using a temperature controller^14,15^. *In vivo* TPM experiments were conducted on the small intestine (jejunum) of live mice by gently pulling the small intestine out of the surgically opened abdominal cavity and by mounting it on the intestinal holder. Gentle pressing of the pulled small intestine with the cover glass helped to reduce the motion artifacts (peristalsis).

For fluorescent labeling of the small intestinal tissue, moxifloxacin (Vigamox, 15 µL, 12.4 mM, Alcon Laboratories), Hoechst 33342 (5 µL, 100 µM, #62249, ThermoFisher Scientific, USA), rhodamine–conjugated Ulex Europaeus agglutinin 1 (UEA-1, 5 µL, 100 µM, #RL-1062, Vector Laboratories, USA), and a lysosomal probe (LysoTracker Red DND-99, 5 µL, 100 µM, #L7528, Invitrogen, USA) were topically administered on the surface of the intact small intestine 10 minutes prior to TPM. In case of TPM imaging from the luminal side (Fig. 1b and Supplementary Video 1) and on the cross-section (Fig. 1d and Supplementary Video 3) of the small intestine, a 5 mm longitudinal incision was made to expose villi of the small intestine during the anesthesia and moxifloxacin was administered onto the lumen 10 minutes prior to TPM.

### Induction and image analysis of Paneth cell degranulation

For TPM of Paneth cell degranulation process in the intact small intestine of live mice *in vivo*, moxifloxacin ophthalmic solution were topically administrated onto the serosa of the small intestine in wild type C57BL/6 SPF mice during anesthesia. Paneth cell degranulation in the small intestine was induced by using CpG-ODNs (5′-TCCATGACGTTC CTGACGTT, #1351325, TIB MOLBIOL, Germany). To monitor the *in vivo* degranulation process of the Paneth cell, CpG-ODNs (0.0636 µg/µL, 30 µL) was careful injected into the lumen of the small intestine. To detected the results of the Paneth cell degranulation, CpG-ODNs (0.4 μg/µL, 100 µL) was intraperitoneally injected 5 hours prior to TPM *in vivo*^27^. As the negative control, PBS solution of the same amount as CPG-ODNs was injected using the same protocol.

The quantitative analysis of Paneth cell degranulation was performed by counting the total number of pixels of Paneth cells granules in the 3D volumetric TP images after intraperitoneal injection. Several image processing algorithms were applied to increase the contrast of Paneth cell granules in TP images (Supplementary Fig. 5). After the image processing, the total number of pixels occupied by Paneth cell granules were counted in 3D TP images using MATLAB (Matlab 2016b, MathWorks). The results were plotted as mean ± standard deviation by using Origin (OriginPro 9.0, OriginLab), and the statistical analysis was performed by Welch’s t-test using Prism (Prism 7, GraphPad). Statistical significance was presented by number asterisks depending on the p-value; ****p < 0.0001.

## Electronic supplementary material

Supplementary Information

Supplementary Video 1

Supplementary Video 2

Supplementary Video 3

Supplementary Video 4

Supplementary Video 5

Supplementary Video 6

Supplementary Video 7

Supplementary Video 8

Supplementary Video 9

Supplementary Video 10

Supplementary Video 11
