# Supplementary material for: Two-photon microscopy of Paneth cells in the small intestine of live mice
Source: Sci Rep. 2018 Sep 21;8:14174. doi: 10.1038/s41598-018-32640-7 (PMC6155010; doi:10.1038/s41598-018-32640-7)
Supplement: Supplementary file 1 — Supplementary Information [file 41598_2018_32640_MOESM1_ESM.docx]

**Supplementary Information**

Two-photon microscopy of Paneth cells in the small intestine of live mice

**Won Hyuk Jang^1,8^, Areum Park^1,8^, Taejun Wang^1^, Chan Johng Kim^1^, Hoonchul Chang^1^, Bo-Gie Yang^2^, Myoung Joon Kim^3^, Seung-Jae Myung^4^, Sin-Hyeog Im^1,5^, Myoung Ho Jang^5^, You-Me Kim^6,*^ & Ki Hean Kim^1,7,*^**

**Affiliations:**

^1^ Division of Integrative Biosciences and Biotechnology, Pohang University of Science and Technology (POSTECH), 77 Cheongam-ro, Nam-gu, Pohang, Gyeongbuk 37673, Republic of Korea

^2^ 507 Avison Biomedical Research Center, Severance Biomedical Research Institute, Yonsei University College of Medicine, 50-1 Yonsei-ro, Seodaemun-gu, Seoul 03722, Republic of Korea

^3^ Department of Ophthalmology, University of Ulsan College of Medicine, Asan Medical Center, 88 Olympic-ro, 43-gil, Songpa-gu, Seoul 05505, Republic of Korea

^4^ Department of Gastroenterology, University of Ulsan College of Medicine, Asan Medical Center, 88 Olympic-ro, 43-gil, Songpa-gu, Seoul 05505, Republic of Korea

^5^ Academy of Immunology and Microbiology, Institute for Basic Science (IBS), 77 Cheongam-ro, Nam-gu, Pohang, Gyeongbuk 37673, Republic of Korea

^6^ Graduate School of Medical Science and Engineering, Korea Advanced Institute of Science and Technology, 291 Daehak-ro, Yuseong-gu, Daejeon 34141, Republic of Korea

^7^ Department of Mechanical Engineering, Pohang University of Science and Technology (POSTECH), 77 Cheongam-ro, Nam-gu, Pohang, Gyeongbuk 37673, Republic of Korea

^8^ These authors contributed equally to this work.

*Correspondence and requests for materials should be addressed to Y.–M. K. (email: youmekim@kaist.ac.kr) and K. H. K. (email: [kiheankim@postech.ac.kr](mailto:kiheankim@postech.ac.kr))

**Contents**

**List of Supplementary Figures**

| **Supplementary Figure 1** | A schematic and a photo of the custom-built intestinal holder for *in vivo* two-photon (TP) imaging of the mouse small intestine |
| --- | --- |
| **Supplementary Figure 2** | *In vivo* label-free TPM of SPF normal, SPF obese (*ob*/*ob*), and germ free (GF) normal mice |
| **Supplementary Figure 3** | 3D reconstruction of TPM images taken from the serosa of the intact small intestine stained with moxifloxacin and rhodamine-UEA-1 in live wild type SPF mice |
| **Supplementary Figure 4** | Fluorescence properties of moxifloxacin |
| **Supplementary Figure 5** | Step-by-step procedure for the quantification of Paneth cell granules in moxifloxacin based TPM images of intestinal crypts |

**List of Supplementary Videos**

| **Supplementary Video 1** | *In vivo* moxifloxacin based TPM video from the luminal side of the small intestine, in a wild type C57BL/6 SPF mouse. |
| --- | --- |
| **Supplementary Video 2** | *In vivo* moxifloxacin based TPM video from the serosa side of the intact small intestine, in a wild type C57BL/6 SPF mouse. |
| **Supplementary Video 3** | *In vivo* moxifloxacin based TPM video from an incised surface of the small intestine, in a wild type C57BL/6 SPF mouse. |
| **Supplementary Video 4** | *In vivo* TPM video from the serosa side of the intact small intestine, in a wild type C57BL/6 SPF mouse, stained with Hoechst 33342, moxifloxacin, rhodamine-conjugated *Ulex Europaeus* agglutinin 1 (UEA-1). |
| **Supplementary Video 5** | *In vivo* moxifloxacin based TPM video of the intestinal crypt in the intact small intestine from the serosa side of a wild type C57BL/6 SPF mouse. |
| **Supplementary Video 6** | *In vivo* autofluorescence (AF) based TPM video of the intestinal crypt in the intact small intestine from the serosa side of a wild type C57BL/6 SPF mouse. |
| **Supplementary Video 7** | *In vivo* moxifloxacin based TPM video of the intestinal crypt of the intact small intestine from the serosa side of an obese (ob/ob) mouse. |
| **Supplementary Video 8** | *In vivo* autofluorescence (AF) based TPM video of the intestinal crypt in the intact small intestine from the serosa side of an obese (ob/ob) mouse. |
| **Supplementary Video 9** | *In vivo* moxifloxacin based TPM video of the intestinal crypt in the intact small intestine from the serosa side of a germ free (GF) normal mouse. |
| **Supplementary Video 10** | *In vivo* autofluorescence (AF) based TPM video of the intestinal crypt in the intact small intestine from the serosa side of a GF normal mouse. |
| **Supplementary Video 11** | 3D reconstruction video of the intact small intestine in a live SPF normal mouse, taken from the serosa side stained with moxifloxacin and rhodamine-UEA-1. |

**Supplementary Figures & Legends**


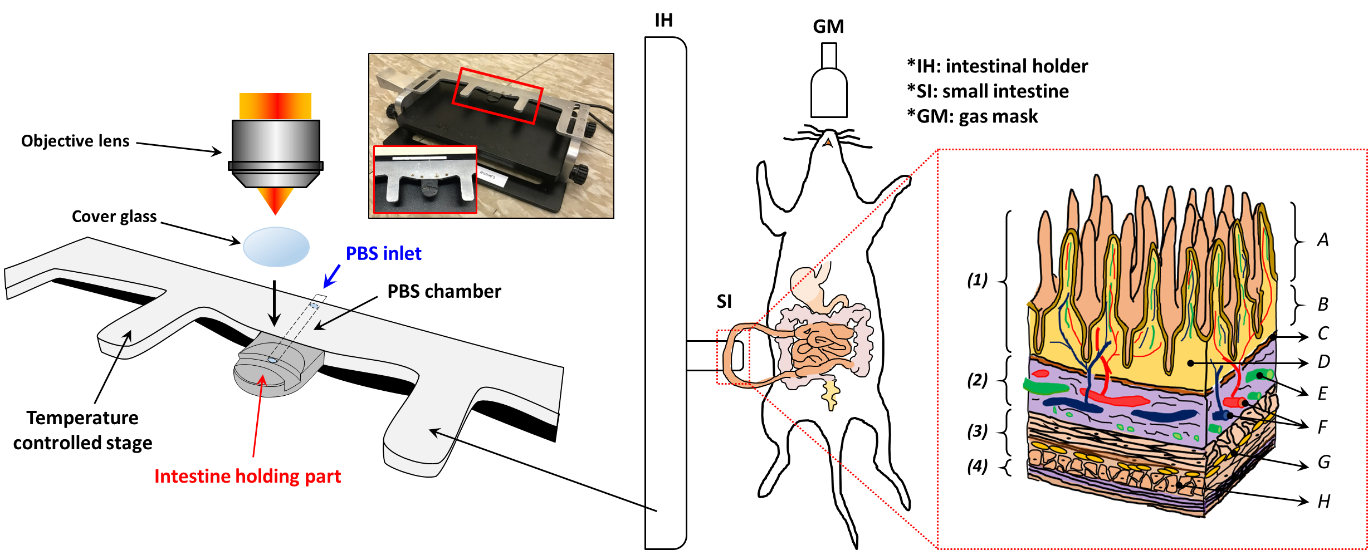


**Supplementary Figure 1:** A schematic and a photo of the custom-built intestinal holder (IH) for *in vivo* two-photon (TP) imaging of mouse small intestine (SI). A mouse was anesthetized with gas mask (GM) and a portion of the SI was gently pulled out from the abdominal cavity and held on the intestinal holder. The cross-section of the mouse small intestine is shown in the illustration. The small intestine consists of four major layers: (1) mucosa, (2) submucosa, (3) muscularis mucosa, and (4) serosa. The detail features of the small intestine are (A) villus, (B) crypt, (C) muscularis mucosa, (D) lamina propria (LP), (E) lymphatic vessel, (F) submucosal artery and vein, (G) myenteric plexus, and (H) longitudinal layer of smooth muscle

**
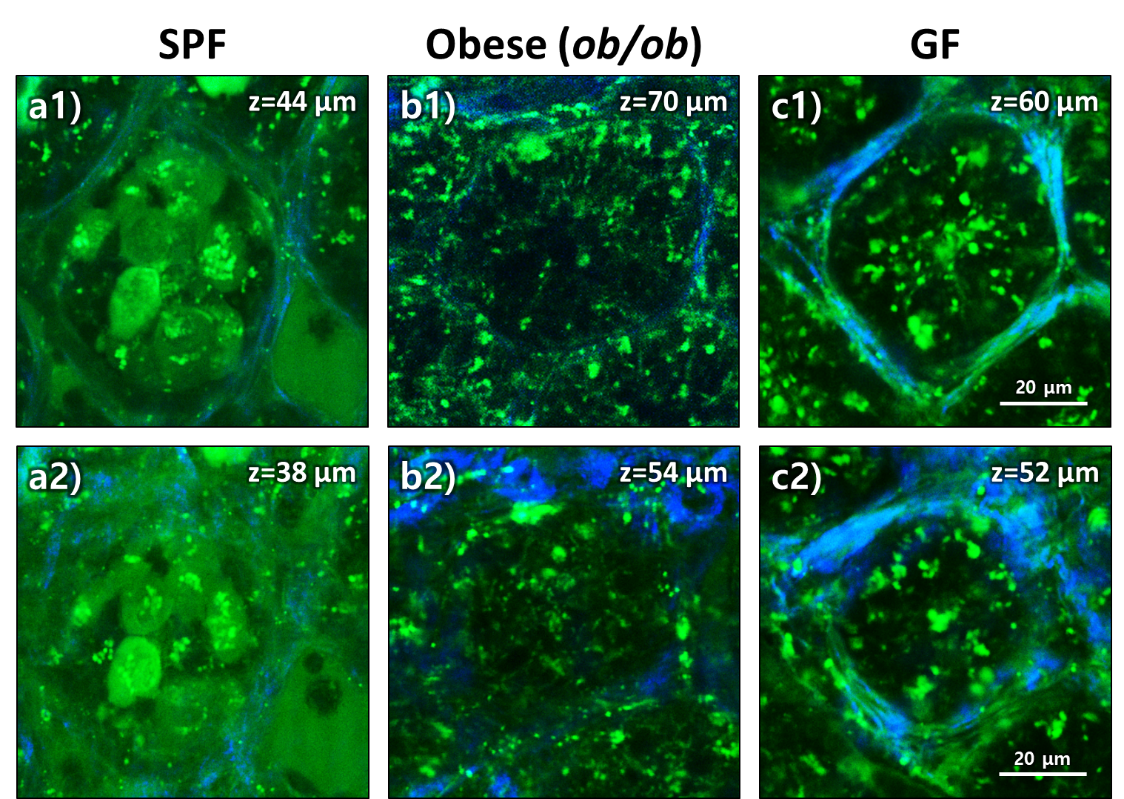
**

**Supplementary Figure 2:** Autofluorescence based TPM images of the intestinal crypt in the small intestine of (a) wild type SPF mice, (b) obese (*ob*/*ob*), (c) and wild type GF mice. (a) AF-based TPM images of Paneth cells in wild type SPF mice. Cellular structures at the base of intestinal crypts were visible by AF expression (**Supplementary Video 6**). Some cells had stronger AF than others, and this variation of cellular AF levels might indicate different intracellular compositions or metabolic activities. There were bright AF expressing spots, some of which were inside AF expressing cells. Because these structures were distributed somewhat random, these might not be Paneth cell granules. (b) AF-based TPM images of Paneth cells in obese (*ob*/*ob*) mice (**Supplementary Video 8**). TPM images did not visualize any distinct cellular structures except scattered bright spots at the base of the intestinal crypts. The invisibility of cellular structures might indicate relatively lower AF expression due to metabolic alteration in obese (*ob*/*ob)* mice compared with lean wild type mice. Boundaries of intestinal crypts were visible via SHG signal from their collagen composition. (c) AF-based TPM images of Paneth cells in GF normal mice (**Supplementary Video 10**). TPM images did not visualize cellular structures based on AF, which was different from TPM images in wild type SPF mice. In contrast, slightly clustered spots expressing bright AF were observed in GF normal mice, similarly to those in SPF mice


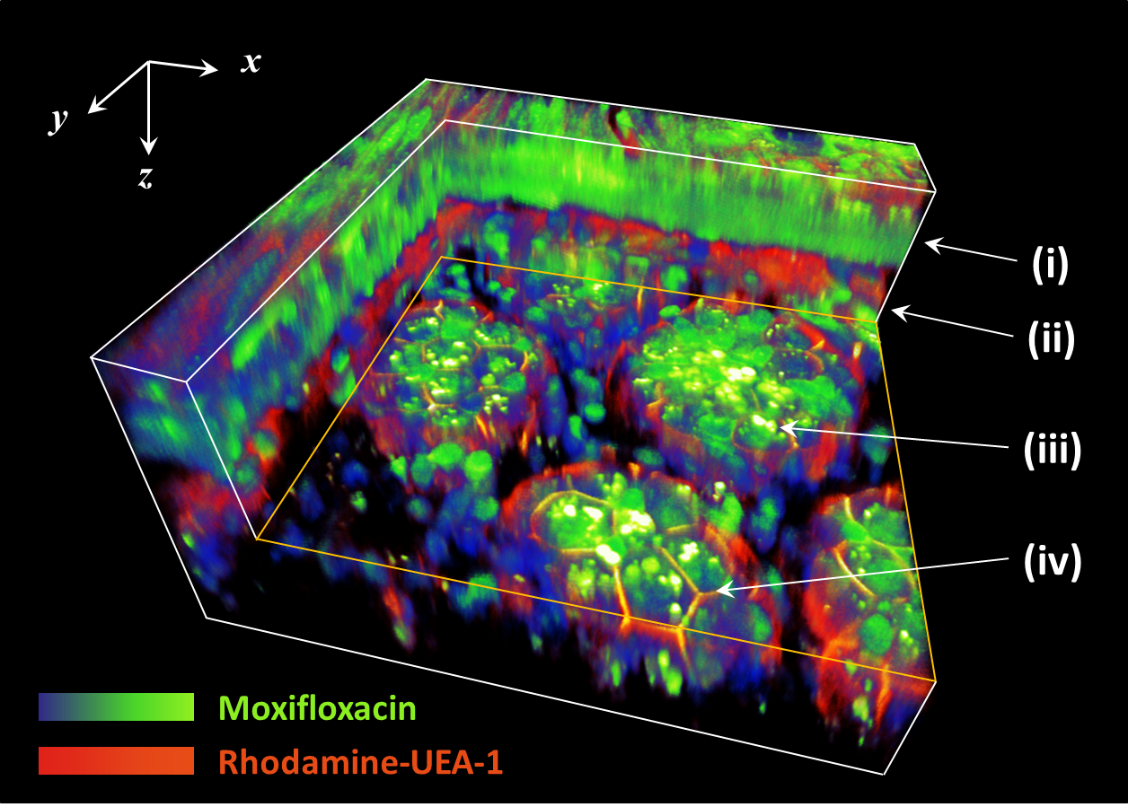


**Supplementary Figure 3**: A 3D reconstructed TPM image of Paneth cells in the intact small intestine labeled with moxifloxacin (volren Green) and rhodamine-UEA-1 (volren Red) in live wild type SPF mice (**Supplementary Video 11**). This 3D image was acquired by scanning from the serosa of the small intestine, and reveals cellular structures of the mouse small intestine between the serosa and the intestinal crypts. The 3D images were reconstructed using 3D data visualization software (Amira 5.3.3., Thermo Fisher Scientific). Moxifloxacin signals are concentrated in the granules of Paneth cells whose membranes are counterstained with rhodamine-UEA-1. Because both moxifloxacin and rhodamine-UAE-1 were topically administered on the serosa of the intact small intestine, they also accumulated in the muscle layer and lamina propria. (i) muscle layer, (ii) lamina propria, (iii) Paneth cell granule, and (iv) Paneth cell membrane.

**
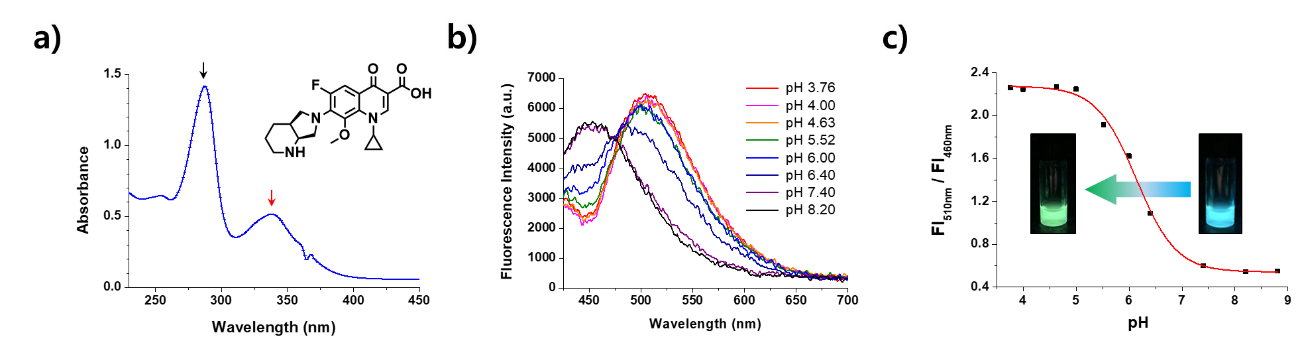
**

**Supplementary Figure 4:** Single-photon fluorescence properties of moxifloxacin. (a) The UV/VIS absorption spectrum of moxifloxacin was measured by using a HP 8453 UV/Vis spectrophotometer. Fluorescence spectrum of moxifloxacin was measured by using a Photon Technology International Fluorescence System (US) with a 1 cm standard quartz cell. The absorption spectrum shows two peaks at approximately 280 nm and 340 nm. (b) The emission spectra of moxifloxacin in various pH conditions from 3.76 to 8.20. The emission spectra were measured by using 380 nm single-photon excitation wavelength. Wavelength shift of the emission peak intensity was observed depending on pH concentration. The peak wavelength was 460 nm at relatively low pH condition, and was shifted to 510 nm at relatively high pH condition. (c) The ratio of fluorescence intensity at the two wavelengths of 510 nm and 460 nm in various pH concentrations. Below pH 5 concentration, fluorescence intensity at 510 nm was higher than the one at 460 nm, and the intensity ratio was approximately 2.2. However, above pH 7 condition, fluorescence intensity at 510 was lower than the one at 460 nm, and the intensity ratio was approximately 0.5. The shift of emission peak wavelength seemed to occur between pH 5 and pH 7.


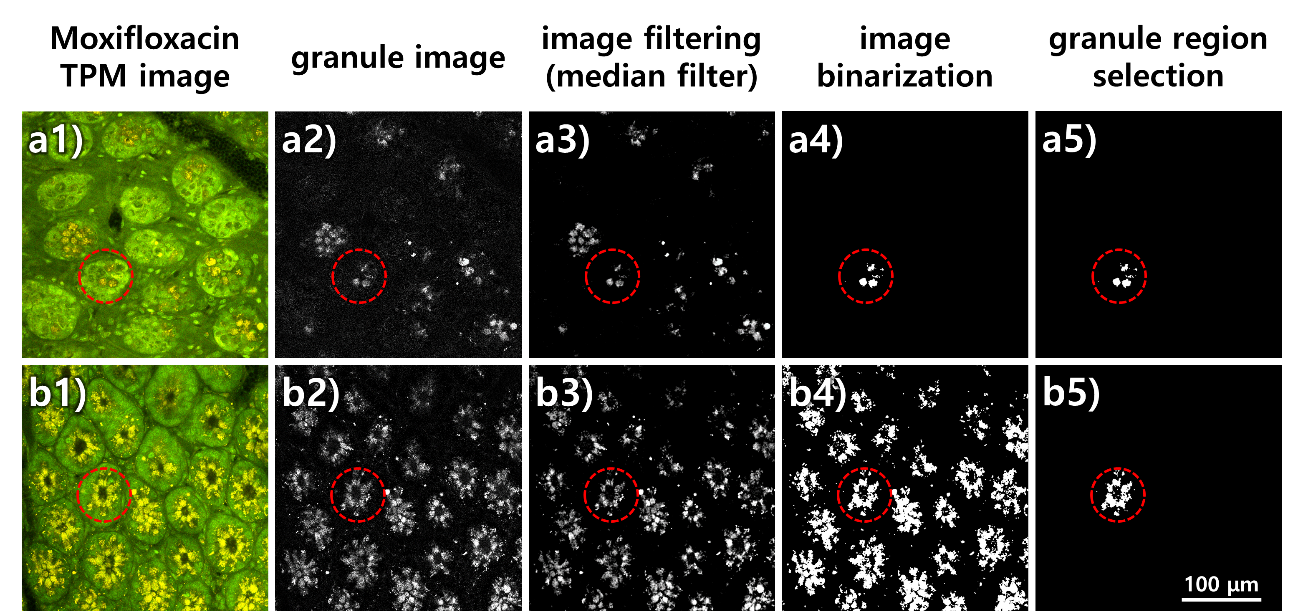


**Supplementary Figure 5**: Image processing procedure for the quantification of Paneth cells granules in the intestinal crypt **(Fig. 3)**. Moxifloxacin based TPM images showed slightly different emission spectra in the cell cytoplasm and granule, owing to the dependency of moxifloxacin emission spectrum on pH condition **(Supplementary Fig. 4)**. Moxifloxacin TPM images **(a1, b1)** were decomposed into 3 color channel images (*red*, *green*, and *blue* channels). Granule images were obtained from the decomposed *green* and *red* channel images **(a2, b2).** The isolated granule images were spatially filtered using 2D median filter for denoising **(a3, b3).** The filtered images were converted into binary images based on thresholding **(a4, b4).** The region of an intestinal crypt was manually selected with a circular mask **(a5, b5)**. The radius of the mask was selected large enough to cover the entire intestinal crypt, but small enough to exclude structures in between the intestinal crypts. The pixel number of Paneth cell granules was counted and summed for series of TP 3D images. This process of region selection and pixel counting was repeated for each intestinal crypt in moxifloxacin based 3D TPM images.

**Supplementary Video & Legends**

**Supplementary Video 1**: *In vivo* moxifloxacin based TPM video from the luminal side of the small intestine in a wild type C57BL/6 SPF mouse. (Corresponding to **Fig. 1(b)**)

The image size consists of 512 × 512 pixels covering a field-of-view (FOV) of 300 μm × 300 μm with a stepwise increment of 2 μm along the z-axis. Imaging speed was 0.78 frames/s and video playback time was 7 second at 5 frames/s rate. Color coding for emission fluorescence was as follows: 300–680 nm (*green*).

**Supplementary Video 2**: *In vivo* moxifloxacin based TPM video from the serosa side of the intact small intestine in a wild type C57BL/6 SPF mouse. (Corresponding to **Fig. 1(c)**)

The image size consists of 512 × 512 pixels covering a FOV of 300 μm × 300 μm with a stepwise increment of 2 μm along the z-axis. Imaging speed was 0.78 frames/s and video playback time was 14 second at 5 frames/s rate. Color coding for emission fluorescence was as follows: 300–495 nm (*green*), 495–680 nm (*red*).

**Supplementary Video 3**: *In vivo* moxifloxacin based TPM video from the incised surface of the small intestine in a wild type C57BL/6 SPF mouse. (Corresponding to **Fig. 1(d)**)

The image size consists of 512 × 512 pixels covering a FOV of 620 μm × 620 μm with a stepwise increment of 2 μm along the z-axis. Imaging speed was 0.78 frames/s and video playback time was 20 second at 3 frames/s rate. Color coding for emission fluorescence was as follows: 300–495 nm (*green*), 495–680 nm (*red*).

**Supplementary Video 4**: *In vivo* TPM video from the serosa side of the intact small intestine in a wild type C57BL/6 SPF mouse, counterstained with Hoechst 33342, moxifloxacin, rhodamine-conjugated *Ulex Europaeus* agglutinin 1 (UEA-1). (Corresponding to **Fig. 1(e)**)

The image size consists of 1024 × 1024 pixels covering a FOV of 70 μm × 70 μm with a stepwise increment of 2 μm along the z-axis. Imaging speed was 0.78 frames/s and video playback time was 5 second at 3 frames/s rate. Color coding for emission fluorescence was as follows: 300–495 nm (*blue*), 495-560 nm (*green*), 560–680 nm (*red*).

**Supplementary Video 5**: *In vivo* moxifloxacin based TPM video of the intestinal crypt of the intact small intestine from the serosa side of a wild type C57BL/6 SPF mouse. (Corresponding to **Fig. 2(a)**)

The image size consists of 512 × 512 pixels covering a FOV of 75 μm × 75 μm with a stepwise increment of 2 μm along the z-axis. Imaging speed was 0.13 frames/s and video playback time was 11 second at 3 frames/s rate. Color coding for emission fluorescence was as follows: 300–680 nm (*green*).

**Supplementary Video 6**: *In vivo* autofluorescence (AF) based TPM video of the intestinal crypt of the intact small intestine from the serosa side of a wild type C57BL/6 SPF mouse. (Corresponding to **Supplementary** **Fig. 2(a)**)

The image size consists of 512 × 512 pixels covering a FOV of 75 μm × 75 μm with a stepwise increment of 2 μm along the z-axis. Imaging speed was 0.13 frames/s and video playback time was 15 second at 3 frames/s rate. Color coding for emission fluorescence was as follows: 300–430 nm (*blue*), 430–680 nm (*green*).

**Supplementary Video 7**: *In vivo* moxifloxacin based TPM video of the intestinal crypt of the intact small intestine from the serosa side of an obese (*ob*/*ob*) SPF mouse. (Corresponding to **Fig. 2(b)**)

The image size consists of 512 × 512 pixels covering a FOV of 75 μm × 75 μm with a stepwise increment of 2 μm along the z-axis. Imaging speed was 0.13 frames/s and video playback time was 13 second at 3 frames/s rate. Color coding for emission fluorescence was as follows: 300–680 nm (*green*).

**Supplementary Video 8**: *In vivo* autofluorescence (AF) based TPM video of the intestinal crypt of the intact small intestine from the serosa side of an obese (*ob*/*ob*) SPF mouse. (Corresponding to **Supplementary** **Fig. 2(b)**)

The image size consists of 512 × 512 pixels covering a FOV of 75 μm × 75 μm with a stepwise increment of 2 μm along the z-axis. Imaging speed was 0.13 frames/s and video playback time was 15 second at 3 frames/s rate. Color coding for emission fluorescence was as follows: 300–430 nm (*blue*), 430–680 nm (*green*).

**Supplementary Video 9**: *In vivo* moxifloxacin based TPM video of the intestinal crypt of the intact small intestine from the serosa side of a germ free (GF) mouse. (Corresponding to **Fig. 2(c)**)

The image size consists of 512 × 512 pixels covering a FOV of 75 × 75 μm with a stepwise increment of 2 μm along the z-axis. Imaging speed was 0.13 frames/s and video playback time was 18 second at 3 frames/s rate. Color coding for emission fluorescence was as follows: 300–680 nm (*green*).

**Supplementary Video 10**: *In vivo* autofluorescence (AF) based TPM video of the intestinal crypt of the intact small intestine from the serosa side of a germ free (GF) mouse. (Corresponding to **Supplementary** **Fig. 2(c)**)

The image size consists of 512 × 512 pixels covering a FOV of 75 μm × 75 μm with a stepwise increment of 2 μm along the z-axis. Imaging speed was 0.13 frames/s and video playback time was 17 second at 3 frames/s rate. Color coding for emission fluorescence was as follows: 300–430 nm (*blue*), 430–680 nm (*green*).

**Supplementary Video 11**: 3D reconstruction video of the intact small intestine in a live wild type SPF mouse, taken from the serosa side counterstained with moxifloxacin and rhodamine-UEA-1. (Corresponding to **Supplementary** **Fig. 3**)

Raw image size consists of 512 × 512 pixels covering a FOV of 150 μm × 150 μm with a stepwise increment of 2 μm along the z-axis and imaging speed of 0.13 frames/s. 3D reconstruction video playback time was 30 second by processing in Amira software. Color coding for fluorescent signals in raw images was as follows: 300–560 nm (*volren Green*), 560–680 nm (*volren Red*).
